# Native American Community Perspectives on Oral Health Access: Understanding the Impact of Rurality

**DOI:** 10.3390/healthcare11202788

**Published:** 2023-10-21

**Authors:** Amanda J. Llaneza, Alex Holt, Lancer Stephens, Julie Seward

**Affiliations:** 1 Southern Plains Tribal Health Board, Oklahoma City, OK 73114, USA; allaneza@spthb.org (A.J.L.); aholt@spthb.org (A.H.); 2 Health Promotion Sciences, Hudson College of Public Health, University of Oklahoma Health Sciences Center, Oklahoma City, OK 73104, USA; lancer-stephens@ouhsc.edu; 3 Oklahoma Shared Clinical and Translational Resources, University of Oklahoma Health Sciences Center, Oklahoma City, OK 73104, USA

**Keywords:** access to oral healthcare, American Indian/Alaska Native, health disparities, oral health prevention, oral health

## Abstract

Purpose: Oral health disparities related to access persist for American Indian/Alaska Native (AI/AN) communities compared to the general population, especially in rural areas of the United States. The objective of this study was to better understand community perspectives of oral health, how rurality impacts access to care, and attitudes towards the implementation of dental therapists in Oklahoma, particularly among the AI/AN population. Methods: A descriptive, observational study design was utilized. An exploratory survey was conducted online and comprised of qualitative and quantitative data. The total frequencies and percentages were evaluated for the quantitative questions. The qualitative data was analyzed using thematic analysis. Utilizing descriptive and qualitative research methods, the focus was to describe the experiences of the respondents and their characteristics related to oral health in Oklahoma. Results: A total of 201 responses were obtained, where 65% (n = 131) identified as an enrolled member or employee of a tribe represented in Oklahoma. Key qualitative themes included community access to care, community concerns, and community motivated solutions. Conclusions: AI/AN communities are an underserved group in healthcare. Although communities in rural areas face major barriers to oral health services, evidence-based solutions can be implemented.

## 1. Introduction

Populations, regardless of racial and ethnic backgrounds, residing in rural areas of the United States (U.S.) often encounter unique and longstanding challenges regarding access to healthcare. According to a 2017 report from the U.S. Census Bureau, over 90% of the land area of the United States is considered rural and about one in five Americans live in rural areas [1]. The issues surrounding healthcare access are further exacerbated for minority populations living in rural America. When investigating racial and ethnic health disparities among rural adults in the United States between 2012 to 2015 with data from the Behavioral Risk Factor Surveillance System (BRFSS) survey, the non-Hispanic Black population (24.5%), Hispanic population (23.1%), and American Indian/Alaska Native (AI/AN) population (19.1%) were unable to see a physician when needed because of the cost of the visit, in comparison to the non-Hispanic White population (15.0%) [2]. In addition to the cost of care, rural and low-income patients face barriers in transportation to appointments. Research on transportation and health status estimates that approximately 25% of lower-income patients cancel or miss appointments due to the lack of transportation [3,4,5,6].

Rurality has a similar effect on access to oral healthcare. In the state of Oklahoma, approximately 34% of the population live in rural communities [7]. In 2022, of Oklahoma’s 77 counties, there were 66 that were considered designated dental health provider shortage areas (dental HPSAs) [8]. Dental HPSAs are areas and/or population groups that are experiencing a shortage of dental providers to sufficiently serve their communities in the United States, according to the Health Resources and Services Administration (HRSA) [9]. For dental HPSA designation, the scoring criteria examines a population-to-provider ratio, the percent of population below 100% of the Federal Poverty Level (FPL), water fluoridation status, and travel time to the nearest source of care outside the HPSA designated area [10]. According to a 2022 report from the American Dental Association (ADA), the numbers of graduates from dental hygienist and dental assistant programs might not be adequate to overcome the burden of personnel who permanently left the profession during the COVID-19 pandemic and those that are expected to retire [11]. Further, the dentist to population ratio decreased by 4.2% in Oklahoma between 2010 and 2020, whereas most states experienced an increase, notably in all the surrounding states [12]. The lack of access to oral health services can have detrimental effects on overall health. In 2000, Surgeon General Dr. David Satcher published the following report: “Oral Health in America: A Report of the Surgeon General” [13]. This report discussed oral health as an integral part of overall health as multiple medical conditions may affect one’s oral health and vice versa [13]. Further, overall health and oral health follow similar patterns in that there is a disproportionate burden of disease that is both treatable and avoidable across the lifespan for minority populations in the United States [14,15]. Seventeen years after this historic publication, Dr. Satcher and Dr. Joyce H. Nottingham remarked on the progress made since that report was first published [16]. These findings were published in the *American Journal of Public Health* titled, “Revisiting Oral Health in America: A Report of the Surgeon General” [16]. This publication noted the evidence of continued disparities among minority groups through the 2011–2012 National Health and Nutrition Examination Survey, albeit without mention of the AI/AN population [16]. Additionally, they noted the importance of a diverse oral health workforce. According to estimates from the National Conference of State Legislatures (NCSL), there are over 750,000 oral health professionals in the United States dental workforce, including dentists, dental hygienists, dental therapists, and community dental health coordinators [17]. Further, HRSA conducted a study of the supply of national-level dentists and dental hygienists in the United States in 2017 and the demand projections by the year 2030 [18]. It was found that there were approximately 147,470 full-time dental hygienists, with a 7% increase in demand for full-time dental hygienists by 2030 [18]. Regarding full-time dentists, there were approximately 190,500 dentists in all dental specialties with a 9% increase in demand by 2030 [18].

The burden of oral disease for the AI/AN population is greater than any other ethnic minority group in the United States [19]. The oral healthcare delivery system for the AI/AN population is complex, as it stems from the overall healthcare system for the AI/AN population, which may include tribally led healthcare, federally led Indian Health Service (IHS) care, or Urban Indian healthcare. Disparities related to the access and health outcomes continue to persist in comparison to the general population of the United States. According to the 2020 census, American Indians and Alaska Natives comprise 2.9% of the U.S. population [20]. Systemic, structural, and historical racism can contribute to oral health disparities [14,15,21], and progress towards equity and systems change halts when the AI/AN population are not represented in the national clinical data reports and/or are racially misclassified [22,23]. The state of Oklahoma comprises the second largest AI/AN population in the U.S. at 16% [20]. The purpose of this study is to better understand community perspectives of oral health, how rurality impacts access to care, and attitudes towards the implementation of dental therapists in Oklahoma, particularly among the AI/AN population. Additionally, the authors commit to increasing the body of work that spotlights the AI/AN community and provide evidence-based recommendations to improve the oral health and overall wellbeing in Indian Country that range from community-based initiatives to systems change approaches.

## 2. Methods

The Southern Plains Tribal Health Board (SPTHB) is a non-profit Tribal public health organization dedicated to serving the 43 tribal nations throughout Oklahoma, Kansas, and Texas. A descriptive, observational study design was utilized. An exploratory survey investigating oral health concerns, attitudes, experiences, and solutions to oral healthcare access and health disparities among Tribal and general populations in the state of Oklahoma was conducted. This was conducted online and contained fourteen quantitative questions and six qualitative open-ended questions. These questions can be found in the Supplemental Table S1. To cast a broad net along the Oklahoma region, which is home to 39 Tribal nations, the survey was shared and distributed through SPTHB social media accounts and SPTHB listserv. The survey was also shared via email to internal staff, SPTHB partners, and professional networks across the state. The eligibility criteria included being a resident of Oklahoma, 18 years of age and older, having a valid email address, and correct spelling of the county of residence. Data collection was conducted between 1 February and 8 March 2021. Residents living in rural areas of Oklahoma were not the original focus or the target population of the survey administration; however, the results highlighted the impact of rurality regarding oral health. The objective of this survey was to utilize descriptive and qualitative research methods to describe the experiences of the respondents and their characteristics related to oral health in Oklahoma. The focus of this research study was to describe the open-ended responses and frequency of variables, without regard to any causal or other hypothesis due to the exploratory nature of the study design [24]. The survey served as a tool to collect data and develop a deeper understanding of the phenomenon under study and describe the results. 

### 2.1. Ethics Approval

Through an exempted review, this manuscript was reviewed and approved by the Oklahoma City Area Indian Health Service Institutional Review Board (IRB) Research and Publication Committee.

### 2.2. Quantitative Data

The quantitative data from this survey were analyzed through descriptive statistics. Participant characteristics that were recorded included occupation, tribe/organization they represent, county of residence, and county of employment. Participants were asked to rank urgent oral health concerns within their communities utilizing a scale of 1 being most urgent, 2 being very urgent, 3 being somewhat urgent, 4 being not so urgent, and 5 being least urgent. This was converted to a categorical variable, where 1 and 2 were combined to represent urgent, 3 remained the same, and 4 and 5 were combined to represent not urgent. Respondents were further asked to identify challenges or barriers for improving oral health within their communities, and to identify suggestions to improve the shortage of dental providers in Oklahoma, all from a pre-set list. Participants were asked about the importance of cultural competence from their oral healthcare providers, as well as knowledge related to dental health aide therapists. The total frequencies and percentages were evaluated for each of the questions. A total of 44 different organizations were represented from a sample of 201 respondents collected. This included Tribal nations, healthcare clinics and agencies, and a category labeled “other”, which consisted of those who did not represent a healthcare clinic or indicate any Tribal affiliation. Most frequencies and percentages were small, except for five categories which consisted of ≥5% of the total sample. To ensure these categories did not have any substantive influence on the overall percentages, each were evaluated. This was performed by separately taking each of these variables out of the dataset to determine if there was a greater than 10% difference in the new percentages for each survey question. Evidence of percent differences between the overall samples when each of the five variables were separately removed was not witnessed. Statistical analysis was performed using SAS, version 9.4 (SAS Institute Inc., Cary, NC, USA).

### 2.3. Qualitative Data

The qualitative data were analyzed through assessing themes in the open-ended response questions. The open-ended response questions allowed the opportunity for participants to further express their experiences with oral health concerns within their communities, as well as identify barriers and possible solutions to addressing concerns that were not already provided in the quantitative questions. Thematic analysis is the process of using a coding system to organize open-ended responses to develop reasonable and meaningful conclusions from the data [25]. The purpose of the open-ended responses was to enhance or confirm the information within the quantitative data. Two researchers (AJL and AH) independently explored the responses from the open-ended questions and generated codes using an inductive and deductive approach. The inductive approach allows for the formation of new ideas and themes to be driven from the data as opposed to the ones produced from the preconceived theory [26]. The deductive approach allows for the building of themes that are constantly checked against the data and the use of both approaches allows for the construction of patterns and themes generated from the collected data [26].

## 3. Results

**Figure 1:**
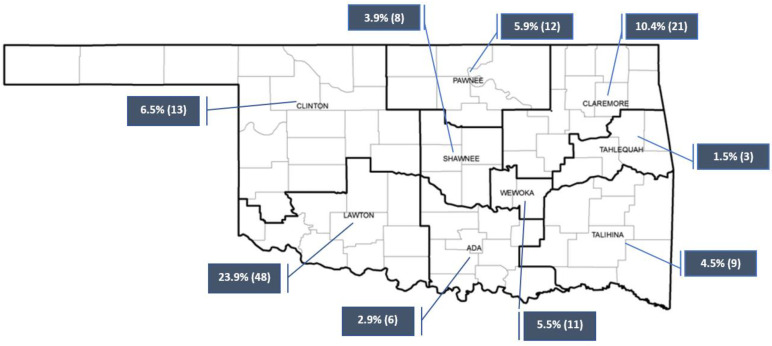
Percentage of respondents in Oklahoma within the Oklahoma Indian Health Service Units, n = 131.

Of the 201 responses collected, 65% (n = 131) identified as an enrolled member or employee of a Tribe represented in Oklahoma. Other than being a member or employee of a Tribe, 28.5% (n = 57) identified as an employee of a healthcare clinic or agency, Tribal agency, academic institution, two or more Tribes, or other. Figure 1 displays the percentage of respondents within the Oklahoma Indian Health Service (IHS) Units among the 131 responses that indicated being a member or employee of a Tribe in Oklahoma. 

**Figure 2:**
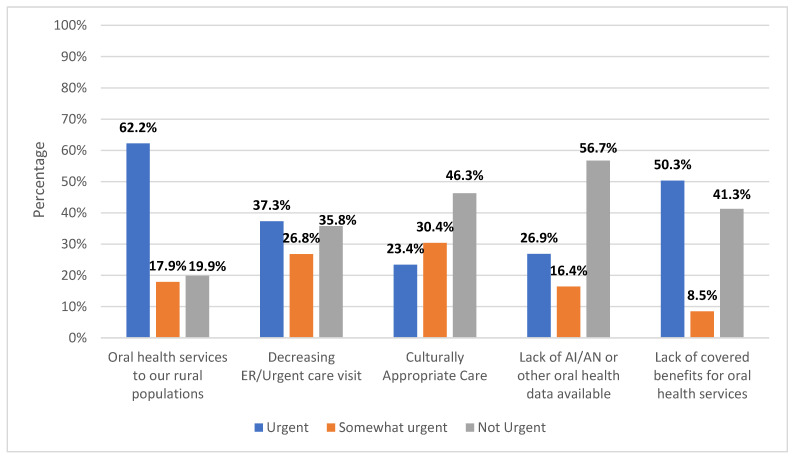
Percentage of oral health concerns of community members ranked from urgent to not urgent, N = 201. ER: emergency room; AI/AN: American Indian/Alaska Native.

When asked to rank oral health concerns within their community from urgent to not urgent, 62.2% of community members ranked oral health services to their rural populations as urgent, and 50.3% ranked the lack of covered benefits for oral health services as urgent (Figure 2). 

Key themes based on the open-ended questions included community access to care, community concerns, and community-motivated solutions. These key themes and categories can be seen in Table 1. 

### 3.1. Community Access to Care

**Figure 3:**
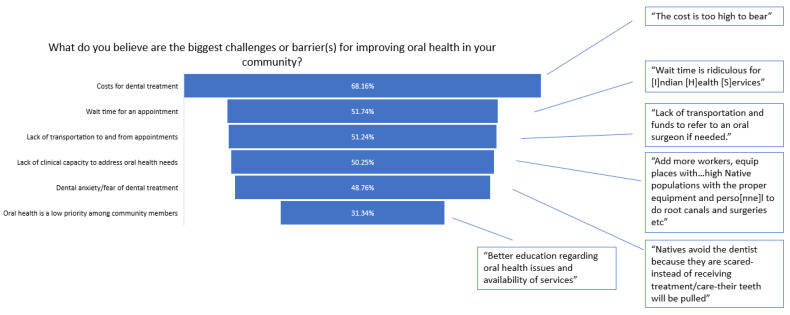
Percentage of challenges or barriers for improving oral health within communities and representative quotes.

When asked to share the biggest challenges or barriers for improving oral health in their communities, 68.16% of respondents selected costs of dental care, 51.74% selected clinic wait times, 51.24% selected lack of transportation, and 50.25% selected clinical capacity (Figure 3), in addition to representative quotes from the open-ended responses. 

Participants were asked to identify any other barriers important to their communities that were not previously listed. Community insights were related to the attitudes on oral health and access, oral health education, clinical practices in their communities, and access to fluoridated water. 

Overwhelmingly, 60.7% of the quantitative responses demonstrated concerns related to dental workforce shortages and lack of available dental services in healthcare clinics within their communities. This was complemented by the open-ended responses where participants voiced concerns over the inability to schedule or receive preventative, restorative, and specialty oral health services among adults in their communities. Respondents also noted an inadequate oral health education and prioritization within their communities, which is especially related to the connection between oral health and overall health. One respondent provided further insight to the community attitudes stating there is a “Lack of understanding how important oral care is and how it can affect more than your teeth and mouth”.

Approximately 80.1% of respondents believed that members of their community would accept appropriate treatment from a licensed dental therapist. For participants who indicated their community members may reject or hesitate to accept treatment from a licensed dental therapist, open-ended responses were encouraged to learn more about community hesitations or concerns. Many of the open-ended explanations indicated a need for better communication and educational awareness about dental therapist occupations, specific procedures they are allowed to perform, and their role in conjunction with the established dental team. One respondent shared, “I would like to know more about dental therapy so that I could make a decision”. Also, 57.9% expressed concerns over the category and class of procedures dental therapists are able to perform, and if the treatments are of similar quality and satisfaction to the same procedures completed by dentists. This was further complemented by open-ended responses where participants indicated concerns about treatments from dental therapists which are normally conducted by dentists. Opened-ended responses also indicated concerns about the Indian Health Service (IHS) or Tribal facilities that hire dental therapists.

### 3.2. Community Concerns

Major concerns voiced by the respondents in opened-ended responses included topics related to the lack of appointment availability, expensive services, cultural relevancy, specialized care for elderly community members, lack of oral health publicity and health education, and prioritization within communities. Regarding cultural relevancy, one respondent shared, “…It doesn’t seem like cultural issues would be important in choosing a dentist, but it comes up more than one would think”. Further, a respondent shared insights to the costs of care related to health education, noting that “There is not enough awareness of oral health and the cost is too high”.

Participants discussed how prolonged wait times for routine or preventative dental services were a major contributing factor in the inability to receive care. For example, one respondent expressed their dissatisfaction regarding lengthy wait times for their scheduled appointment, noting that the “Wait time is ridiculous for [I]ndian [H]ealth [S]ervices” (Figure 3). Lastly, respondents expressed concerns for older adults within their communities and their oral health where one participant indicated that “Elderly oral health problems are more prominent”.

### 3.3. Community Motivated Solutions

Participants provided solutions to oral health challenges related to children in their community, adults in their community, rural communities, medical providers, and educational awareness. Solutions provided an insight into the importance of patient-informed decisions for community members. These solutions included increasing the access to services, adherence to health recommendations, establishing oral health services as part of routine general healthcare, and offering early care information for new parents. For children, community members indicated that offering new parents early care information would improve health, as well as offering school-based clinical outreach, oral health educational demonstrations in schools, and integrate more pediatric dentists in rural communities. One respondent shared their insights for improving oral healthcare for children and stated, “I think providing service school based can be the best option for children and providing education starting in grade school about oral health”. For adults in the community, solutions included oral health services covered by Medicaid to help reduce the burden of medical costs.

Many participants voiced frustration regarding the lack of available specialty oral health treatment within their communities. Participants indicated that they have “no oral surgeons or specialists,” and the burden of needing to “travel 55 miles east to see any specialist”. To increase the access to services, some respondents suggested that family medicine, primary care, or general practitioners should be educated in basic oral healthcare. One respondent stated, “In this community it is important for healthcare providers to be trained in all area[s] due to [the] lack of physicians in Indian Health Service”.

Lastly, participants noted educational awareness is a viable solution for improving oral health within their communities. One such response stated, “I believe a large scale TV or internet campaign about how oral health is part of overall healthcare may help. In our area, the majority appear to think it is not related to other chronic issues and it has been proven that good oral health can help prevent some chronic issues”. Further aspects of educational awareness included the use of social media and the local news for oral health education campaigns. 

## 4. Discussion

Residents of rural areas in the United States face unique challenges in obtaining access to oral healthcare. Further, the “Oral Health in America: Advances and Challenges,” a report published in 2021, reiterated that minority communities in the United States and those living in poverty experience worse oral health outcomes compared to the general population [27]. With approximately one third of Oklahomans living in rural areas, our findings contribute further evidence to the lack and increased need of oral health services in rural areas of Oklahoma.

A lack of oral health providers in rural areas of the United States is a major driver in exacerbating oral healthcare access and further widens the inequities of oral health in minority populations. Since the publication of the report from the surgeon general in 2000, national calls to action to address oral health disparities have been pursued. Such recommendations include diversifying the oral health workforce personnel and capacity. Expanding the oral health workforce is a proven viable solution for many communities. It is evident from the results that further education is needed for community members to understand the capabilities of expanded workforce members, such as dental health aide therapists (DHAT) or dental therapists. The results above indicated the need for better communication and education about dental therapists. This is a matter of health literacy, and, as is known, it is intermediate to low among most adults in the United States [28]. A media campaign is called for with clear information on dental health aide therapists, including their training and scope of service in order to reduce the population’s medical fear of the unknown. 

Community members may not have adequate knowledge about dental therapists and could think that, due to historical underfunding for tribal health services, they would have to “settle” for a mid-level provider. In reality, dentists lead the oral health team and provide supervision for dental therapists, dental hygienists, and dental assistants. Dentists are trained to perform an array of procedures, while dental therapists focus on the most needed procedures, primarily in provider shortage areas and community-based settings [29] The dental therapy workforce has proven successful in rural communities and contributes to reducing oral health workforce shortages. For example, findings from a study investigating dental therapists working in the Alaska Native communities in the Yukon-Kuskokwim Delta found that dental therapy treatment days were positively associated with preventive care utilization and negatively associated with extractions for children and adults [30].

Findings from observational quantitative studies on dental utilization in rural communities served by dental therapists highlight the importance and efficacy of this provider [31,32]. To improve access to oral healthcare in Oklahoma, incorporating dental therapists is one viable answer. As established earlier, respondents expressed major concerns related to the shortages of the oral health workforce and the demand for specialty dental services as well as restorative care within their communities. In addition, community members expressed a lack of understanding of the services that dental therapists can provide, attributing to why they may not desire to be treated by one. The costs or lack of insurance and payment options are other reasons why community members may reject or hesitate to receive care from a licensed dental therapist. The scope of work for dental therapists varies state to state but generally includes nonsurgical extractions, filling cavities, and the placement of temporary crowns [33]. Considering services provided by dental therapists can relieve dentists from overbearing workloads, resulting in these dentists becoming readily available to perform intricate and complicated, revenue-generating treatments. A study in Minnesota concluded that dental therapists practicing in two various clinics increased revenue for each clinic and improved access to care for the patients [34]. In addition, considering that dental therapists are less expensive to hire compared to dentists, dental practices have the opportunity to provide more services for Medicaid patients and still be profitable, despite the low reimbursement rates [35].

Although communities in rural areas face major barriers to oral health services, evidence-based solutions can be implemented. Such solutions for improving care and addressing disparities include the access to dental health coverage included in medical insurance, broadening the oral health workforce, improving transportation availability and access to affordable, income-based dental services. As evidenced from the results, routine care can be particularly cumbersome if access to services results in lengthy wait times for appointments. The pre-pandemic dentist-to-population ratio at the national Indian Health Service level was 1 dentist per 2830 AI/AN patients, in the Indian Health Service Oklahoma City Service Area, it was 1 dentist per 3210 AI/AN patients [17]. In comparison, the ratio was 1 dentist per 2018 patients for the general population of Oklahoma [36]. 

Another chief concern is that the AI/AN health practitioner representation is often not reported. Research has indicated the importance of racial and ethnic minority representation in healthcare and indicated that patients are more likely to participate in routine care and have more positive experiences and interactions with the healthcare system when healthcare practitioners are of the same racial/ethnic background as them, and understand their cultural needs [37,38]. According to the American Dental Association, in the state of Oklahoma, 82.7% of practicing dentists are White, 4.9% are Asian, 3.9% are Black, 1.7% are Hispanic, and there was no information reported for dentists who identify as AI/AN [39]. With Oklahoma having the second largest population of AI/AN in the nation, educational institutions and workforce recruitment initiatives should focus on diversity and inclusion.

### Limitations

The primary limitation of an exploratory study design is that there is no evidence of a temporal relationship. As all of the variables were collected simultaneously, it is not possible to determine any causal inferences. The focus of this research was exploratory and it aimed to describe oral health concerns, attitudes, experiences, and community-driven solutions for the access of oral health in Oklahoma, especially for Tribal populations, and no sample size calculations were performed. The results are limited, as significance tests were not performed due to the descriptive nature of the study design. This study was also limited in the demographic variables that were collected, and the ability to determine the response rate during the data collection phase. Data collection took place over a relatively short period of time, which limited the data collection compared to a longer period. Participation in the survey was voluntary, which may have resulted in unintended bias. This study only collected data from respondents from the state of Oklahoma, which may impact the generalizability of the results compared to the broader U.S. population. 

## 5. Conclusions

Residents of rural areas in the United States face continued challenges with regard to the access of oral health services. These challenges include long wait times for appointments, lack of dental insurance coverage and/or acceptance from providers, and even finding providers in their area. Such barriers are further exacerbated for minority communities living in rural areas. AI/AN communities are an underserved group in healthcare, and improving healthcare access and health outcomes is a matter of improving health inequities. However, the solutions offered are attainable, and, in some instances, planning or implementation is already underway. Incorporating and centering community voices in health policy and public health initiatives is imperative to sustainable change, progress, and general awareness. The results from this survey provides further insight into the concerns and potential solutions for the improved access to dental healthcare, especially for the populations in rural areas. Expanded efforts should be considered to incorporate community voice into advocacy and further action towards improved oral health for all Oklahomans. 

## Figures and Tables

**Table 1:** Table of themes and categories from the open-ended responses.

Theme	Category	Representative Quote(s)
**Community Access to Care**		
Barriers		
	1. Attitudes of oral health	“Lack of understanding how important oral care is and how it can affect more than your teeth and mouth”.
	2. Oral health education	
	3. Clinical practices in community	
	4. Access to fluoridation	
Dental Therapy		
	5. Acceptance of treatment from a dental therapist	“I would like to know more about dental therapy so that I could make a decision”.
	6. Improve educational awareness of dental therapy	
**Community Concerns**		
	7. Lack of appointment availability	“…It doesn’t seem like cultural issues would be important in choosing a dentist, but it comes up more than one would think”.“There is not enough awareness of oral health and the cost is too high”.“Elderly oral health problems are more prominent”.
	8. Expensive services	
	9. Cultural relevancy	
	10. Specialized care for elderly community members	
	11. Lack of oral health educational awareness	
	12. Lack of oral health prioritization within communities	
**Community Motivated Solutions**		
Children populations		
	13. School-based clinical outreach	“I think providing service school based can be the best option for children and providing education starting in grade school about oral health”.
	14. Oral health education demonstrations in schools	
	15. Pediatric dentists in rural communities	
Adult populations		
	16. Inclusion of oral health services covered by Medicaid	“Medicaid expansion for adults. Oral health care is important but can be expensive”.
Medical providers		
	17. Lack of specialty oral health treatment within communities	“In this community it is important for healthcare providers to be trained in all area[s] due to [the] lack of physicians in Indian Health Service”.
	18. Important to train general practitioners in basic oral health	
Educational awareness		
	19. Use of social media	“I believe a large scale TV or internet campaign about how oral health is part of overall healthcare may help. In our area, the majority appear to think it is not related to other chronic issues and it has been proven that good oral health can help prevent some chronic issues”.
	20. Use of the local news	

## Data Availability

The data presented in this study are available upon request from the corresponding author. The data are not publicly available to protect the privacy of the subjects.

## Supplementary Materials

The following supporting information can be downloaded at: https://www.mdpi.com/article/10.3390/healthcare11202788/s1, Table S1. Questionnaire.
