# Supplementary material for: Native American Community Perspectives on Oral Health Access: Understanding the Impact of Rurality
Source: Healthcare (Basel). 2023 Oct 21;11(20):2788. doi: 10.3390/healthcare11202788 (PMC10606298; doi:10.3390/healthcare11202788)
Supplement: Supplementary file 1 [file healthcare-11-02788-s001.zip › healthcare-2617229-supplementary.pdf]

Supplemental File
Survey of Oral Health Challenges and Solutions among the Native and General Population of Oklahoma
Supplemental Table S1. Questionnaire.

1. What is your title/occupation?
2. What tribe/organization do you represent?
3. What is your county of residence?
4. In what county do you currently work?
5. Please rank the following from the most urgent to the least urgent oral health concerns for your community. 1- Most urgent; 2-Very urgent; 3- Somewhat urgent; 4-Not so urgent; 5-Least urgent

    Oral health services to our rural population
    Decreasing emergency room/urgent care visits
    Culturally appropriate care
    Lack of American Indian/Alaska Native or other oral health data available
    Lack of covered benefits for oral health services

6. Does your community have any other oral health concerns, other than those mentioned above? *(opened-ended response)* ____
7. What do you believe are the biggest challenges or barrier(s) for improving oral health in your community? *Select all that apply*.

    Lack of clinical capacity to address oral health needs
    Lack of transportation to and from appointments
    Costs for dental treatment
    Wait time for an appointment
    Dental anxiety/fear of dental treatment
    Oral health is a low priority among community members
    Other (please specify) _____________________________________

8. According to Health Resources and Services Administration (HRSA), in 2020 almost 85% of counties in Oklahoma lack a sufficient number of oral health providers to properly serve their communities. Given this shortage of dental providers, what are some suggestions on how to address this issue? *Select all that apply.*

    Scholarship/loan repayment options
    Teledentistry
    Expand dental workforce
    Expand scope of practice for dental hygienists
    Recruit dental providers from underserved communities
    Other (please specify) ___________

9. What are some suggestions on how to improve oral health overall in Oklahoma (*i.e. school based health centers, Medicaid expansion for adults, expanding dental workforce, dental therapy, policy changes, etc.)?* If no suggestions, write "none" in box.

____

10. How important is it for you to receive care from someone that understands your community/culture?

    Extremely important
    Very important
    Somewhat important
    Not so important
    Not at all important

Dental Health Aide Therapists (also called dental therapists) are dental care providers similar to nurse practitioners or physician assistants in medicine. They practice under the supervision of a dentist and are able to deliver routine dental care to patients. These providers have been used in Alaska Native communities since 2004.

11. How would you rate your knowledge level on dental therapy as a profession?

Very knowledgeable
Somewhat knowledgeable
Slightly knowledgeable
Not knowledgeable

12. Given your general knowledge, do you believe that dental therapy is a practical solution for Oklahoma?

Yes
No
I don't know/unsure

13. If no, why don't you believe dental therapy is a practical solution for Oklahoma?

____

14. Would you encourage tribal/organizational leaders to publicly support dental therapy as one solution to improve access to oral health treatment and prevention services in your community?

Yes
No
Maybe

15. If no, please explain why.

____

16. Has your tribal government considered incorporating dental therapists into your tribal health system?

Yes
No
I don't know/unsure
Not applicable

17. Do you think people in your community would be willing to receive treatment from a dental therapist?

Yes
No
I don't know/unsure

18. If no or unsure, why don't you believe those in your community would be willing?

____

19. Are you interested in more information/education about dental therapy?

Yes
No

20. Please list any other concerns, questions, or comments you may have about improving oral health in Oklahoma.

____
